# Ultra-High Molecular Weight Polyethylene Modifications Produced by Carbon Nanotubes and Fe_2_O_3_ Nanoparticles

**DOI:** 10.3390/polym15051169

**Published:** 2023-02-25

**Authors:** Alfio Torrisi, Lorenzo Torrisi, Mariapompea Cutroneo, Alena Michalcova, Milena D’Angelo, Letteria Silipigni

**Affiliations:** 1 Dipartimento Interateneo di Fisica, Università di Bari Aldo Moro, 70125 Bari, Italy; 2 Istituto Nazionale di Fisica Nucleare (INFN), Sezione di Bari, 70126 Bari, Italy; 3 Department of Mathematics and Computer Sciences, Physical Sciences and Earth Sciences (MIFT), University of Messina, 98166 Messina, Italy; 4 Nuclear Physics Institute of the CAS, Hlavní 130, 250 68 Husinec–Řež, Czech Republic; 5 Department of Metals and Corrosion Engineering, University of Chemistry and Technology, 166 28 Prague, Czech Republic

**Keywords:** ATR-FTIR, carbon nanotubes, energy gap, Fe_2_O_3_ nanoparticles, UHMWPE, UV–Vis

## Abstract

Thin sheets of ultra-high molecular weight polyethylene (UHMWPE), both in pristine form and containing carbon nanotubes (CNTs) or Fe_2_O_3_ nanoparticles (NPs) at different concentrations, were prepared. The CNT and Fe_2_O_3_ NP weight percentages used ranged from 0.01% to 1%. The presence of CNTs and Fe_2_O_3_ NPs in UHMWPE was confirmed by transmission and scanning electron microscopy and by energy dispersive X-ray spectroscopy analysis (EDS). The effects of the embedded nanostructures on the UHMWPE samples were studied using attenuated total reflectance Fourier transform infrared (ATR-FTIR) spectroscopy and UV–Vis absorption spectroscopy. The ATR-FTIR spectra show the characteristic features of the UHMWPE, CNTs, and Fe_2_O_3_. Concerning the optical properties, regardless of the type of embedded nanostructures, an increase in the optical absorption was observed. The allowed direct optical energy gap value was determined from the optical absorption spectra: in both cases, it decreases with increasing CNT or Fe_2_O_3_ NP concentrations. The obtained results will be presented and discussed.

## 1. Introduction

Ultra-high molecular weight polyethylene (UHMWPE) is a biocompatible polymer which, thanks to its interesting properties, can be employed as a high-performance material in several fields, such as biomedicine [1], engineering [2], microelectronics [3], chemistry, and physics [4,5,6,7]. In fact, it has good chemical and physical stability, mechanical resistance, a low friction coefficient, low wear volume, high crystallinity, high ultimate tensile strength, high biocompatibility [8], and high thermal conductivity [9]. Polymers such as UHMWPE have applications in microelectronics, e.g., as insulators in many electronics components, and in biomedicine, e.g., to realize organ-on-chip microsystems, where cells growing over polymeric membranes are used in simulations of biological barriers and transport processes [10], or as prostheses, e.g., as the femoral head and its acetabular cup [11]. With UHMWPE, it is also possible to create fixed and mobile joints for bones, substrates for cell cultures [12], thin foils to reduce wear during surface friction [13], materials resistant to water and saline solutions [14], lightweight high-strength biocompatible and water equivalent materials useful for radiotherapy research [15], or, by incorporating high hydrogen and deuterium content, it can be used to prepare peculiar samples useful for nuclear research [16]. Such applications can be improved by enhancing the properties of UHMWPE, for example by increasing its chemical and physical stability, its mechanical resistance, its wear resistance, and its biocompatibility. This because the properties of UHMWPE, such as its wetting ability, its electrical and thermal conductivities, and its optical properties, can be modified and controlled using a low percentage of nanoparticles embedded in the polymer, as is reported in the literature [17,18,19,20,21].

However, oxidative degradation due to the generation of free radicals may occur if UHMWPEs are irradiated with ionizing radiation [22]. This problem can be overcome by employing innovative methodologies using bulk and surface modification techniques that can enhance some of the properties of UHMWPE [23]. Moreover, UHMWPE modifications can be produced by innovative treatments using ion implantation techniques and ion, electron, X-ray and laser beam irradiations, which, by inducing hydrogen degassing, enable the realization, in a controlled manner, of electrically conductive tracks in the insulating polymer [24,25,26,27].

Because of their size, shape, and concentration, carbon nanotubes (CNTs) represent a promising choice for modifying UHMWPE in a controlled manner. Due to their exceptional properties, such as their high elastic modulus and tensile strength [28], it is possible to improve the mechanical polymer behavior and thus produce UHMWPE nanocomposites with better mechanical properties. Adding a small amount (~0.2 wt.%) of single-wall carbon nanotubes (SWCNTs) to UHMWPE is enough to increase its hardness and elastic modulus by ~66% and ~58%, respectively [29], and to significantly modify its optical and electrical properties [30,31]. However, the effective utilization of CNTs in combination with UHMWPE to produce nanocomposites is strongly dependent on their uniform dispersion in the matrix, which preserves their structural integrity. A high concentration of CNTs makes UHMWPE dark and highly absorbent to visible light, UV, and IR radiations [32]. 

UHMWPE wear durability and resistance are also impressively improved by various other types of fillers [33], such as nanoparticles (NPs) of gold, titanium, silicon, silver, and iron oxide (Fe_2_O_3_) with diameters in the order of 10–100 nm, even at concentrations lower than 1% in weight. In particular, gold, titanium, silicon, and silver NPs have been embedded in the polymer to change its electrical and thermal conductivities, optical absorbance, mechanical resistance, and elasticity [34,35,36]. With respect to iron oxide (Fe_2_O_3_) nanoparticles, they can be incorporated into the UHMWPE matrix to modify its color, transparency, surface, and bulk properties, enhancing its absorption of UV–Vis and IR radiations [37]. However, it is possible to effectively tailor the properties of the final UHMWPE nanocomposite by controlling the in-depth profile, the concentration, size, and shape of the NPs embedded in the polymer. 

In this work, we have studied the modifications of 50% crystalline UHMWPE produced by adding CNTs and Fe_2_O_3_ nanoparticles. For this purpose, we have used ATR-FTIR spectroscopy to investigate how the UHMWPE chemical bonds change. Moreover, employing UV–Vis spectroscopy, we have investigated the optical absorption coefficient of the thin sheets of UHMWPE as a function of the type and concentration of the embedded nanostructures. Scanning and transmission electron microscopies and energy dispersive X-ray spectroscopy analysis have allowed us to verify the presence of the embedded nanostructures. 

## 2. Materials and Methods

**Figure 1:**
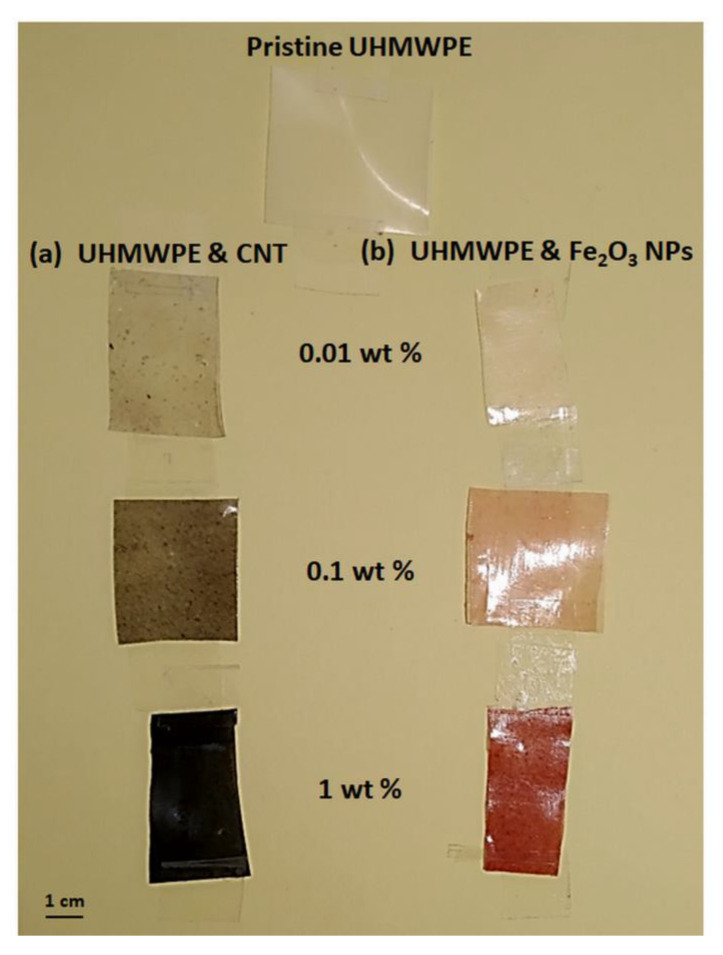
Photos of some of the prepared UHMWPE samples: pristine and (**a**) with different CNT concentrations and (**b**) with different Fe_2_O_3_ NP concentrations.

UHMWPE resin Ticona-GUR 1020 (ρ = 0.93 g/cm^3^, Mw ≈ 3 × 10^6^ g/mol) was employed as a polymer. The matrix was filled with the powder of multi-walled carbon nanotubes (from Sigma Aldrich, Darmstadt, DE, Europe [38]), having diameters of 6–13 nm and lengths of 2.5–20 μm, or with iron oxide nanoparticles (from Sigma Aldrich, Darmstad, DE, Europe), having sizes in the order of ~50 nm [39], both with a purity level of 99.999%. Nanocomposites were made by mixing the UHMWPE with different weight percentages (from 0.01% to 1%) of the CNTs (named, for simplicity, as “UHMWPE & CNT”), employing 99.8% pure ethanol (from Fluka Chemical Corporation, Ronkonkoma, NY, USA) as a dispersing means. More details about the preparation can be found in [23,31]. The UHMWPE & Fe_2_O_3_ nanocomposites were also prepared by mixing the UHMWPE with different weight percentages of Fe_2_O_3_ NPs ranging from 0.01 to 1 weight %, with final density values within the (0.935–1.018) g/cm^3^ range. Both of the mixtures were kept in an ultrasound bath at room temperature (25 °C/2 h). The solvent was then separated under stirring in a heated plate and molded in a hot press at 200 °C for 20 min (at a pressure of about 20 MPa), obtaining sheets of about 50 mm × 50 mm in surface and 100 μm in thickness by releasing Teflon substrates. The pristine UHMWPE sheets had a semitransparent appearance, the UHMWPE & CNT sheets were black, while the UHMWPE & Fe_2_O_3_ sheets were red, as is shown in Figure 1, in which a set of photos of some of the prepared UHMWPE samples with different CNT (a) and Fe_2_O_3_ NP (b) concentrations is shown. The thicknesses of the prepared samples were measured using a micrometer. Their structural properties were evaluated using a Fourier transform infrared (FTIR) spectrometer (Jasco Mod. 4600, Jasco-Europe, Lecco, IT, Europe) equipped with an ATR (attenuated total reflectance) accessory in the (400–4000) cm^−1^ wavenumber range. Their optical properties were also investigated using a double-beam UV–Vis–nIR spectrophotometer (Jasco Mod. V-750, Jasco-Europe, Lecco, IT, Europe) in the (300–900) nm wavelength range. The obtained characterizations were compared with those of the pure UHMWPE.

The surface morphology, size, shape, and dispersion level of the nanostructures embedded in the UHMWPE were microscopically investigated. A scanning electron microscope (SEM, Abingdon, Oxfordshire, UK) by Oxford instruments equipped with a TVIPS camera, operating in the secondary-electron mode at an acceleration voltage of 5 kV on areas of 25 μm × 25 μm, was employed. To obtain a clearer image of the structures embedded in the UHMWPE matrix, further transmission electron microscopy (TEM) and scanning TEM-coupled energy dispersive X-ray spectroscopy (STEM-EDS) analyses were carried out. Specifically, TEM micrographs of the samples were obtained using a Jeol JEM-2200FS (Jeol, Akishima, Japan) field emission electron microscope equipped with an in-column energy filter. To enhance the image contrast and assist in the localization of elements of interest in the sample, the three windows method at a 250 eV, 270 eV, 294 eV energy shift and a 20 eV energy slit was adopted [40]. For the STEM-EDS analysis, a small volume of the active material suspended in ethanol was drop-cast onto a lacey carbon Cu grid. The Cu signal obtained in the EDS spectra due to the X-rays emitted by the Cu grid hit by backscattered electrons was not reported.

## 3. Results and Discussion

### 3.1. ATR-FTIR Spectroscopy

**Figure 2:**
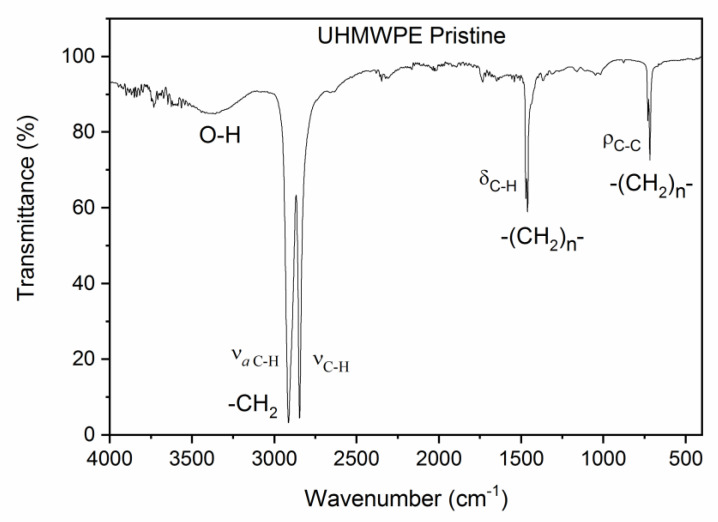
Pristine UHMWPE ATR–FTIR spectrum.

The attenuated total reflectance (ATR) accessory coupled to the FTIR spectrometer (Jasco-Europe, Lecco, IT, Europe), under normal atmospheric conditions, without vacuum, facilitated the evaluation of the UHMWPE IR transmittance vs. the wavenumber, as is shown in the spectrum of Figure 2, which is characterized by two sharp absorption peaks at about 2908 cm^−1^ and 2846 cm^−1^ due, respectively, to the C–H asymmetric and symmetric stretching vibrations in the –CH_2_– group. The spectrum also displays two smaller absorption peaks at 1454 cm^−1^ and 718 cm^−1^. The peak at 1454 cm^−1^ can be attributed, in agreement with the literature [41], to the C–H deformation vibrations in –(CH_2_)_n_–, while the peak at 718 cm^−1^ is due to the C–C rocking vibrations in –(CH_2_)_n_–. Figure 2 also shows that a weak broad band appears between 3600 cm^−1^ and 3250 cm^−1^; this structure can be attributed to the O–H stretching vibrations.

**Figure 3:**
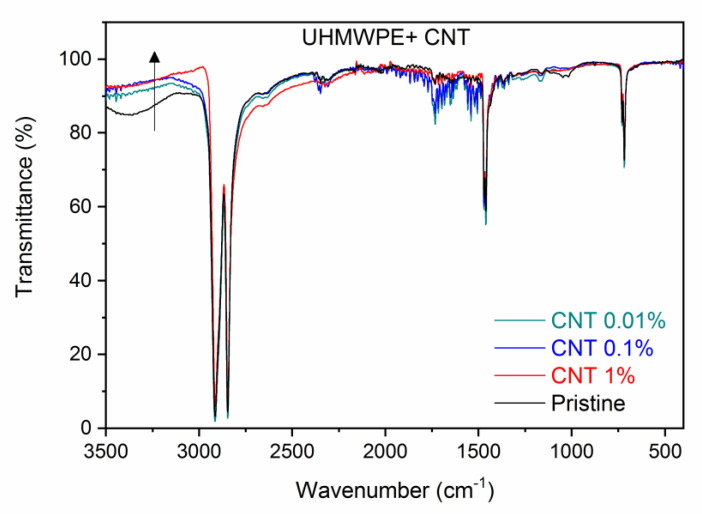
ATR–FTIR spectra comparison between the pristine UHMWPE and its composites with CNTs at different weight concentrations. The up arrow indicates the transmittance increment at high wavenumbers as CNTs concentration increases.

**Figure 4:**
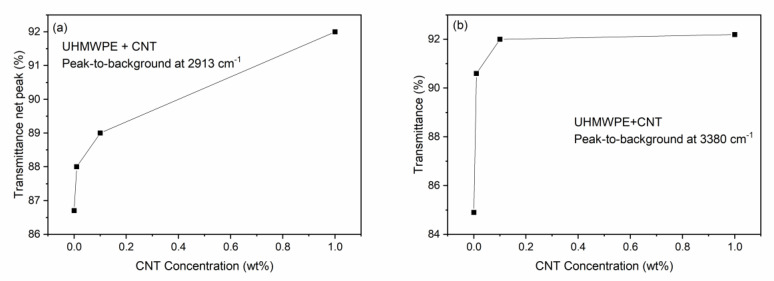
(**a**) Transmittance peak at 2913 cm^–1^ and (**b**) transmittance at 3380 cm^–1^ vs. CNT concentration.

Figure 3 shows the ATR-FTIR spectra comparison between the pristine UHMWPE and the UHMWPE containing CNTs at weight concentrations of 0.01%, 0.1%, and 1.0%. Small differences in the spectra exist due to the different concentrations of embedded CNTs, but the characteristic vibrational peaks of the UHMWPE remain fairly constant. At higher wavenumbers, it is possible to observe that the transmittance increases with the CNT concentration (see up arrow). For instance, at 3500 cm^−1^, the transmittance is 85%, 90%, 91%, and 92.5% for CNT concentrations of 0%, 0.01%, 0.1%, and 1%, respectively. Moreover, a detailed analysis of the main characteristic peaks of the UHMWPE shows that the transmittance peak-to-background ratio increases with the CNT concentration. In fact, the plot of the main peak-to-background at 2913 cm^−1^ vs. the CNT concentration increases, as is shown in Figure 4a. Thus, the embedded CNTs do not substantially modify the main UHMWPE vibrational peaks and make the polymer more transparent in the IR region. The band between 3600 cm^−1^ and 3250 cm^−1^, attributed to the O–H vibrations, decreases in intensity with the CNT concentration, indicating a higher transmittance in the polymer containing the nanotubes, according to the plot shown in Figure 4b, relative to the transmittance values at 3380 cm^−1^.

Due to the low concentration of the employed CNTs, the FTIR spectrum does not show typical vibrational structures because there are carbon nanotubes in the region of the investigated wavenumbers. The characteristic CNT vibrational peaks reported in the literature around 3302 cm^−1^, 2941 cm^−1^, and 1654 cm^−1^ [42], are indistinguishable from the background in our spectra. Thus, the transmission peaks caused by the C–H stretching vibrations in the CH_2_ group remain in their wavenumber positions, but their peak-to-background intensity increases with the concentration of CNTs embedded in the UHMWPE. Moreover, even if we do not reveal any of the CNTs’ characteristic IR features, probably due to their diameters being too large or their concentrations being too low, as indicated in the literature [43,44], we observe a decrease in IR absorption in the polymer nanocomposites at these wave numbers as the CNT concentration increases. 

**Figure 5:**
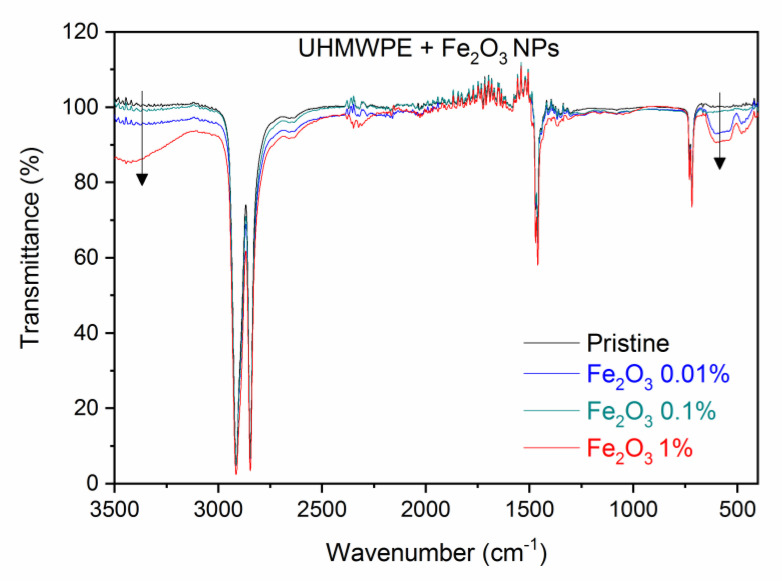
ATR–FTIR spectra comparison between the pristine UHMWPE and its composites with Fe_2_O_3_ nanoparticles at different weight concentrations. The down arrows indicate the O-H (on the left) and Fe-O (on the right) bands intensity increment as Fe_2_O_3_ NPs concentration increases.

Figure 5 shows the ATR-FTIR spectra comparison between the pristine UHMWPE and the UHMWPE containing Fe_2_O_3_ at weight concentrations of 0.01%, 0.1%, and 1.0%. Besides the characteristic peaks of the UHMWPE, one can observe below 700 cm^−1^ (in the so-called fingerprint region) some features around 600 cm^−1^ that can be attributed, in agreement with the literature [45], to the Fe–O stretching mode. These features become more and more pronounced as the Fe_2_O_3_ nanoparticle concentration increases (down arrow on the right). The IR transmission is strongly reduced with the presence of embedded Fe_2_O_3_ nanoparticles. The band associated with the O–H vibration around 3430 cm^−1^ increases in intensity with the concentration of embedded NPs (down arrow on the left). 

**Figure 6:**
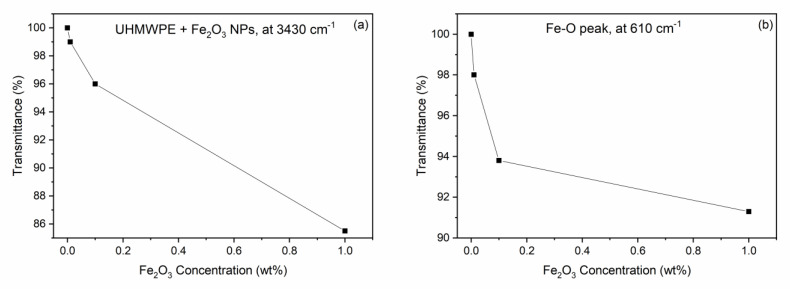
Transmittance decreasing in the UHMWPE as a function of the Fe_2_O_3_ NP concentration at about (**a**) 3430 cm^–1^ and (**b**) 610 cm^–1^.

As with the CNTs, a quantization can be performed by evaluating the transmittance decrease in the peaks at about 3430 cm^−1^ and 600 cm^−1^ due to the O–H and Fe–O stretching vibrations, respectively, as a function of the Fe_2_O_3_ nanoparticle concentration. Figure 6a shows the transmittance value of the large band at about 3430 cm^−1^ as a function of the Fe_2_O_3_ weight concentration, indicating a significant transmission decrease with the Fe_2_O_3_ NP concentration. Figure 6b shows the decrease in the transmittance at the large peak located at about 610 cm^−1^ with the Fe_2_O_3_ NP weight concentration. Both plots indicate that the IR absorption increases with the presence of the Fe_2_O_3_ NPs in the UHMWPE.

### 3.2. UV–Vis Spectroscopy

**Figure 7:**
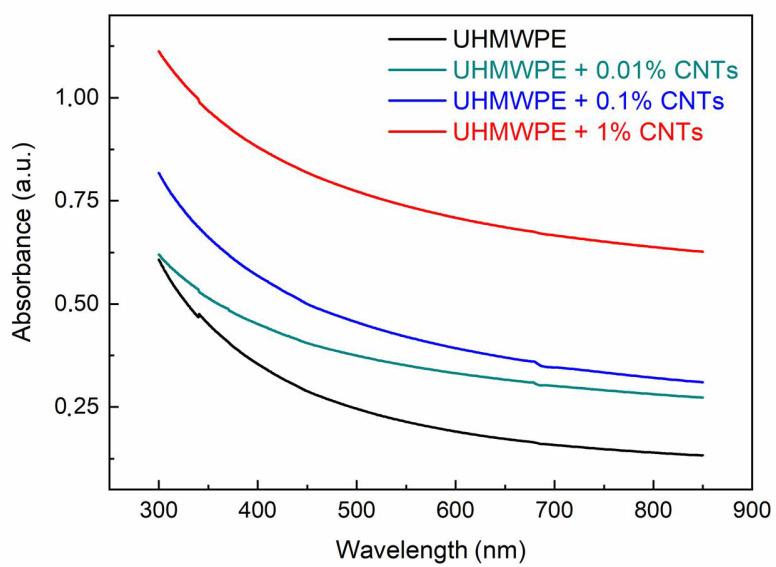
UV–Vis absorbance spectra vs. wavelength of the UHMWPE with embedded CNTs at different weight concentrations.

**Figure 8:**
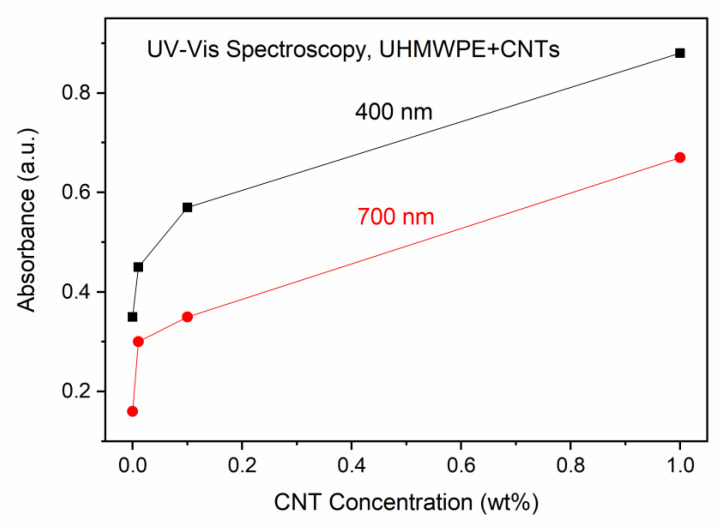
Absorbance in UHMWPE vs. CNT concentration at 400 nm and 700 nm wavelengths.

Figure 7 shows the UV–Vis absorption spectra of the UHMWPE with embedded CNTs at different weight concentrations. Unlike the IR radiation, the absorption in the near UV and visible regions increases with the CNT concentration, a finding which is in agreement with the literature, and with the color change in the samples, which become more and more black and absorbent [46]. The absorbance increment depends on the CNT concentration and light wavelength. A comparison of the absorbance at 400 nm and 700 nm vs. CNT concentration is presented in Figure 8.

Because the optical absorption analysis gives information about the band structure of a given material, it is possible to calculate the UHMWPE optical energy gap (*Eg*) from its optical absorption spectra using the following Mott and Davis relation [47]:

*α hν* = *B*(*hν* − *Ε_g_*)*^n^*, (1)

where *α* and *hν* are the absorption coefficient and incident photon energy, respectively, *B* is a constant, *Eg* is the value of the optical energy gap between the valence band and the conduction band, and the exponent *n* is an empirical index that characterizes the involved transition type. In fact, the *n* exponent takes the value of 1/2, 2, 3/2, and 3 for allowed direct, allowed indirect, forbidden direct, and forbidden indirect transitions, respectively. 

**Figure 9:**
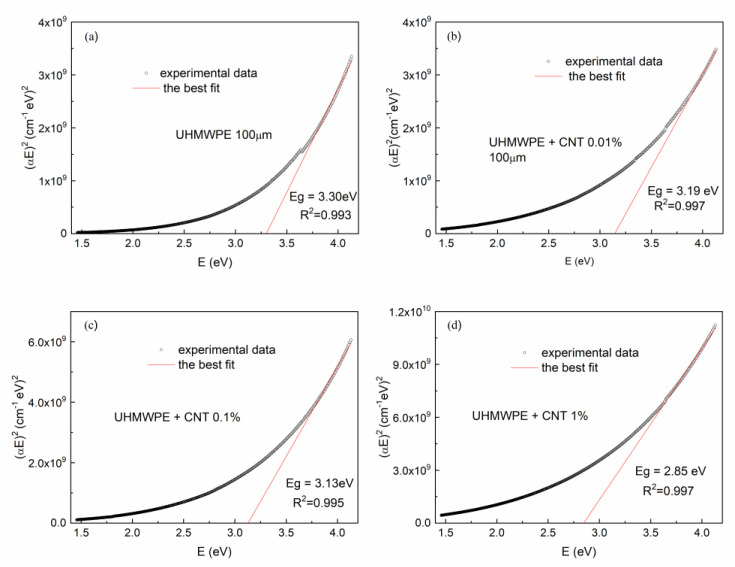
(αE)^2^ vs. photon energy E for (**a**) the pristine UHMWPE and (**b**–**d**) the UHMWPE containing CNTs at different concentrations.

For the determination of the direct optical *Eg*, (*αE*)^2^ has been plotted as a function of photon energy E. The plots for the pristine UHMWPE sample and for those containing CNTs at different concentrations are shown in Figure 9a–d respectively. The *α* coefficient has been calculated in terms of the Beer–Lambert law as follows:

*α* [cm^−1^] *=* 2.303 *A/d*

where *d* is the thickness of the sample and *A* is its optical absorbance. 

The value of the optical energy gap *Eg* has been calculated taking into account the linear portion of the fundamental absorption edge of the UV–Vis spectra plotted in Figure 9, extrapolating it and finally determining the intersection of the extrapolated line with the photon energy axis. The obtained optical energy gap *Eg* values are indicated in Figure 9. 

As is shown in Figure 9a, the pristine UHMWPE exhibits an optical band gap of about 3.30 eV, which is in agreement with the literature [48], while the UHMWPEs containing CNTs at different concentrations have optical energy gaps that decrease with the increasing CNT concentrations, ranging from 3.19 eV for 0.01% to 2.85 eV for 1%. The four plots of Figure 9 indicate the R^2^ coefficient values relative to the best fit of the linear portion of the (*αE*)^2^ vs. E curve used for the optical energy gap evaluation.

**Figure 10:**
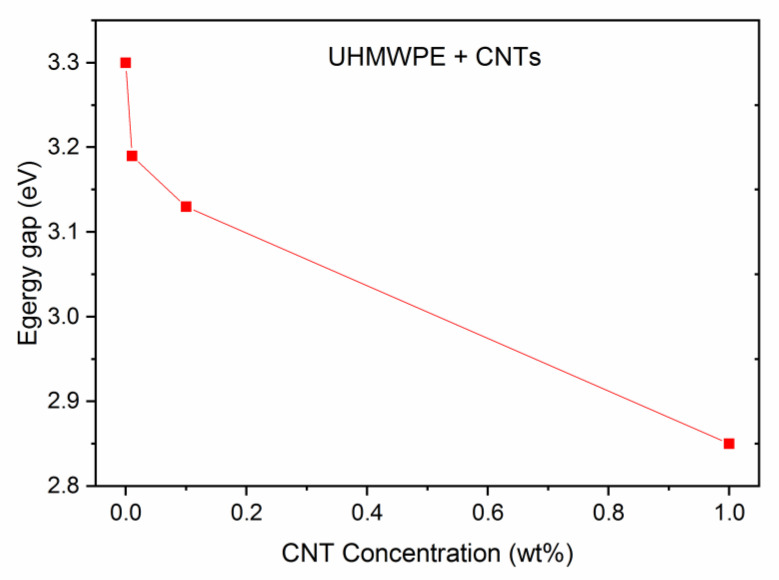
Energy gap vs. CNT concentration in UHMWPE.

Figure 10 depicts the deduced decreasing trend of the optical energy gap *Eg* vs. the CNT concentration in the UHMWPE. It decreases from 3.30 eV (for the pristine sample) to about 2.85 eV (for the sample containing 1 wt% CNTs). This decrease in the optical energy gap values may be attributed to the presence of new electronic levels due to the embedded nanostructures within the energy band gap. In fact, it is well known that carbon atoms, carbon aggregates, and carbon clusters are supposed to be rich with charge carriers that enhance electrical conductivity and, consequently, also influence the optical properties of such materials [49]. The decrease in the optical energy gap implies an increase in the electrical conductivity of the UHMWPE polymer containing the CNTs. 

**Figure 11:**
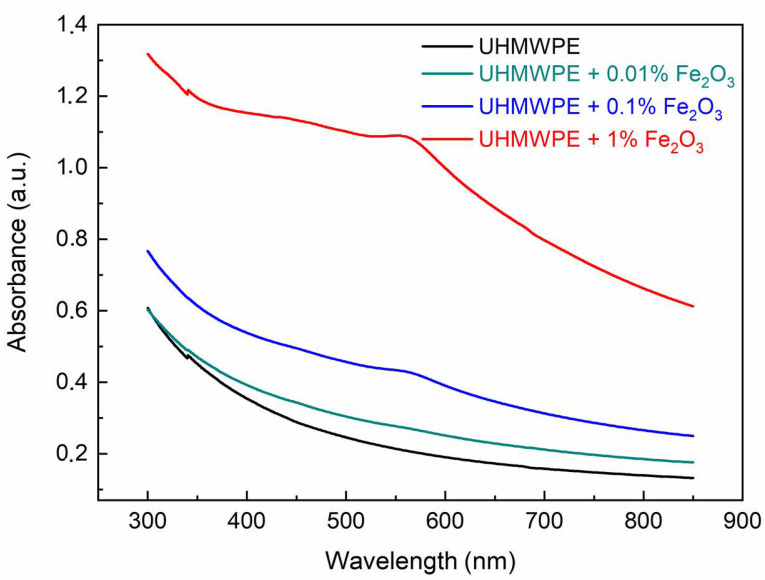
UV–Vis absorption spectra of the UHMWPE with embedded Fe_2_O_3_ NPs at different concentrations.

**Figure 12:**
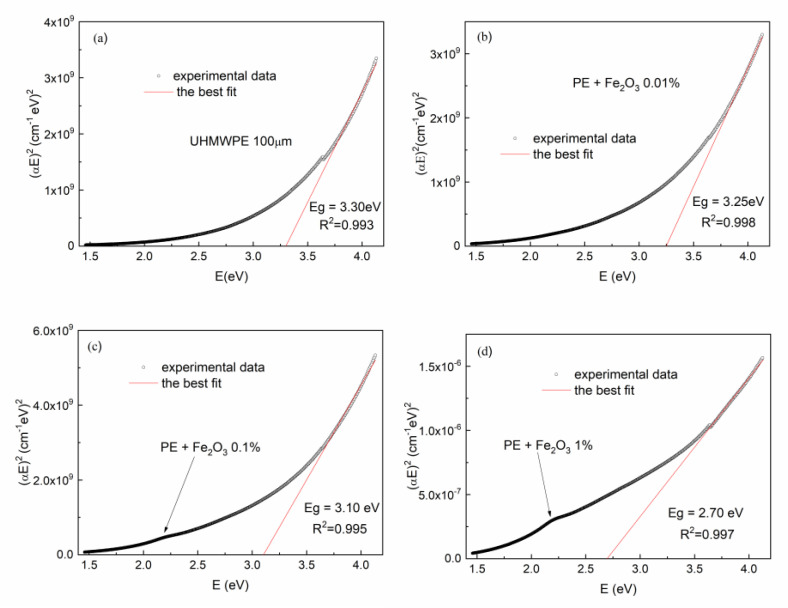
UV–Vis absorption spectra of the UHMWPE with embedded Fe_2_O_3_ NPs at different concentrations from 0 (**a**) up to 1.0 (**b**–**d**).

Figure 11 shows the UV–Vis optical absorption spectra of the UHMWPE with embedded Fe_2_O_3_ NPs at different concentrations. In this case, the optical absorbance increases with the Fe_2_O_3_ NP concentration. As with the UHMWPE reinforced with CNTs, the direct optical energy gap *Eg* value for the UHMWPE containing Fe_2_O_3_ NPs at different concentrations has been deduced from the (*αE*)^2^ vs. E curves (Figure 12).

Even the UHMWPE containing Fe_2_O_3_ NPs at different concentrations has an optical energy gap that decreases with increasing Fe_2_O_3_ NP concentrations ranging from 3.25 eV for 0.01% to 2.70 eV for 1.0%. The weak broadband absorption that already appears at a concentration of 0.1% in the region between 2.0 eV and 2.5 eV (indicated with an arrow in Figure 12c,d) can be assigned, according to the literature [50], to quasi-spherical and/or polyhedral-shaped Fe_2_O_3_ nanoparticles with an average diameter of 75 nm, and could arise from the Laporte forbidden d–d transitions of Fe^3+^ cation in the octahedral coordination site [50]. The four plots of Figure 12 indicate the R^2^ coefficient values relative to the best fit of the linear portion of the (*αE*)^2^ vs. E curve used for the optical energy gap evaluation.

**Figure 13:**
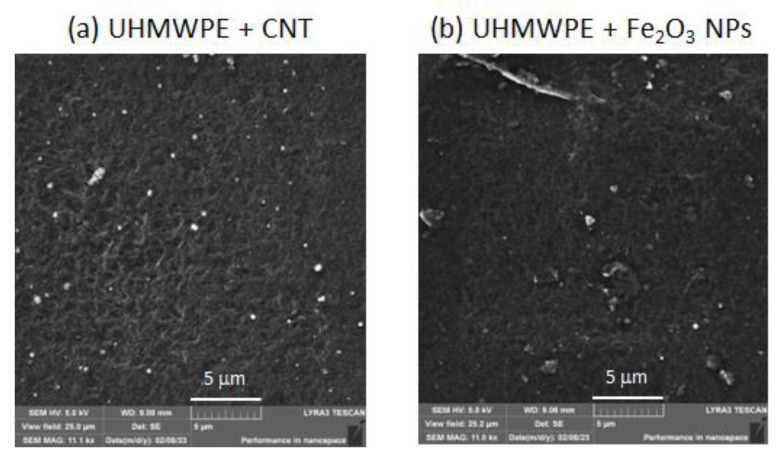
SEM images of the UHMWPE surface (**a**) with CNTs and (**b**) with Fe_2_O_3_ NPs embedded.

The morphological examination of the CNT and Fe_2_O_3_ structures embedded in the UHMWPE polymeric matrix was performed using SEM. Images of the UHMWPE/CNT and UHMWPE/Fe_2_O_3_ nanocomposites with magnifications of 11.1 kX and with a view field of 25 μm are shown in Figure 13a,b, respectively. In both cases, the images reveal small aggregates of nanoparticles on their surfaces whose average size is about 100 nm or less.

**Figure 14:**
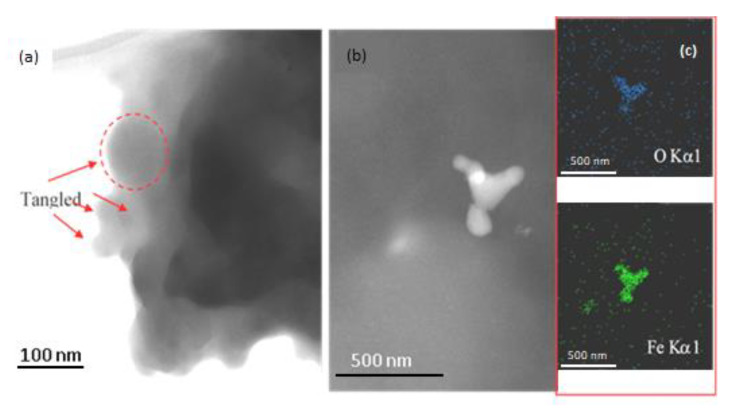
Images of (**a**) the tangled CNT and (**b**) Fe_2_O_3_ agglomerates in the UHMWPE polymer matrix obtained using TEM. In (**c**), the STEM-EDS maps for the Fe_2_O_3_/UHMWPE nanocomposite reveal the presence of Fe and O.

In the TEM micrograph (Figure 14a), several CNTs tangled in the UHMWPE polymeric matrix have been indicated by red arrows, and a more evident big agglomerate of CNTs with a diameter of about 80 nm has been marked with a dashed red circle. The appearance of the tangled CNTs suggests their sub-optimal dispersion. Typically, a state of complete dispersion is achieved by optimizing the dispersion protocol, especially during the breakdown step, which is necessary to avoid the entangling of CNTs. 

In Figure 14b, a TEM micrograph of the UHMWPE/Fe_2_O_3_ nanocomposite indicates the presence of Fe_2_O_3_ aggregates with sizes up to about 200 nm. To identify the presence of Fe and O in the nanocomposite, elemental maps of X-ray characteristic fluorescence from Fe (6.4 keV) and O (525 eV) Kα lines were collected, as is shown in the (c) insert. 

Differential scanning calorimetry (DSC) analysis of the pristine UHMWPE and the polymer containing 1 wt% CNTs and Fe_2_O_3_ NPs are in progress. The preliminary results confirm the data in the literature, i.e., that the UHMWPE melting temperature of about 138 °C increases to 139 °C and 140 °C with the inclusion of CNTs and Fe_2_O_3_ NPs, respectively [23,51,52], indicating a slightly higher thermal stability in the obtained nanocomposites. 

## 4. Challenges and Future Outlook

UHMWPEs modified by nanoparticles and nanostructures represent an important challenge for the realization of innovative polymeric materials with peculiar physical and chemical properties. UHMWPE is a biocompatible material with excellent insulating properties and chemical stability. It represents one of the lightest and most resistant materials on the market, with a high Young’s modulus under different stresses, high wear resistance, and low permeability. It has high resistance to acids, alcohols, bases, esters, petrol, fats, and oils. UHMWPE is used in many fields, from biomedicine to microelectronics, and from engineering to chemistry. 

The synthesis of nanocomposite materials using nanoparticles embedded into UHMWPE can enhance some of its properties and obtain not only more mechanically resistant, harder, and stronger polymers, but also polymers that absorb more light and ionizing radiations. The embedding of nanostructures in UHMWPE can improve the welding and the wetting properties of UHMWPE with other substances, modify its permeability, and modify the amount of carbon, oxygen, hydrogen, and other elements in its composition. Specifically, the UHMWPE+CNTs nanocomposite has been employed to improve the welding process with other polymers, while the UHMWPE+Fe_2_O_3_ nanocomposite has been employed to enhance glass transition temperature, as has been reported in previous investigations.

In the future, it will be possible to modify only some parts of the polymer, realizing conductive tracks, printing electrical resistance and capacitance in the UHMWPE substrates. It will also be possible to embed microelectronic devices in UHMWPE and to make more efficient biomedical prostheses with greater durability and reliability, meeting the demand for prostheses to replace bone joints subjected to high pressure loading.

## 5. Conclusions

In this paper, we have studied the effects of reinforcing UHMWPE with CNTs and Fe_2_O_3_ NPs on its structural and optical properties using ATR-FTIR and UV–vis spectroscopies. The morphological examinations of the CNTs and Fe_2_O_3_ structures embedded in the UHMWPE using SEM revealed, in both cases, small aggregates of nanoparticles with average sizes of about 100 nm or less. The presence of the embedded nanostructures was also revealed through the ATR-FTIR spectra at the highest used concentration, and no significant vibrational change in the UHMWPE structure was observed. In the IR region, a decrease in the absorption was measured as the CNT concentration grew, while an increase in the absorption was measured using the Fe_2_O_3_ NPs. For both embedded nanostructures, the allowed direct optical energy gap value *Eg* of the corresponding UHMWPE nanocomposite was calculated from the relative optical absorption spectrum. 

Our study shows that, at the employed concentrations of CNTs or of Fe_2_O_3_ NPs, and at temperatures during the nanocomposite preparation not higher than 200 °C, the embedded nanostructures do not significantly change the UHMWPE structure. Their main effect consists in introducing new electronic levels within the UHMWPE energy bandgap. This presence affects the UHMWPE’s optical properties, reducing its optical energy gap as the CNT or of Fe_2_O_3_ NP concentration increases. 

The employment of UHMWPE as a composite polymer represents an open challenge due to the different problems that are to be overcome in its use. The dispersion of nanoparticle fillers represents a difficult challenge due to its high viscosity. By increasing the nanoparticle concentration, the surface/volume ratio can be increased, and the surface properties of the nanoparticles become a major factor in influencing its interfacial properties and its mechanical, optical, and electrical behavior. The results presented above can be used to employ the obtained UHMWPE nanocomposites in applications in which it is necessary to increase optical absorbance and electrical conductivity. In contrast, the possibility of varying the IR absorption by embedding CNTs or Fe_2_O_3_ NPs may be useful in modifying the IR laser absorption in thin UHMWPE foils to control the laser melting effect and their welding to different interfaces. Finally, the reduction of the energy gap can be used to increase the electrical conductivity of the UHMWPE or to generate electron traps in the polymer band gap, thus modifying its insulator-like band structure.

## Data Availability

Not applicable.

## References

[1] Patil N.A., Njuguna J., Kandasubramanian B. (2020). UHMWPE for biomedical applications: Performance and functionalization. Eur. Polym. J..

[2] Chang B.P., Akil H.M., Nasir R.M. (2013). Mechanical and Tribological Properties of Zeolite-reinforced UHMWPE Composite for Implant Application. Procedia Eng..

[3] Gao C., Lu H., Ni H., Chen J. (2017). Structure, thermal conductive, dielectric and electrical insulating properties of UHMWPE/BN composites with a segregated structure. J. Polym. Res..

[4] Spiegelberg S., Kozak B.S.A., Braithwaite G., Kurtz S.M. (2016). Characterization of Physical, Chemical, and Mechanical Properties of UHMWPE. UHMWPE Biomaterials Handbook.

[5] Torrisi L., Cutroneo M., Calcagno L., Rosinski M., Ullschmied J. (2014). TNSA ion acceleration at 10^16^ W/cm^2^ sub-nanosecond laser intensity. J. Phys. Conf. Ser..

[6] Torrisi L., Torrisi A. (2023). Shockwave and spallation in silver and other materials by sub-ns laser pulse at 10^16^ W/cm^2^ intensity. Contrib. Plasma Phys..

[7] Torrisi L., Cutroneo M., Torrisi A., Silipigni L., Costa G., Rosinski M., Badziak J., Wołowski J., Zaraś-Szydłowska A., Parys P. (2019). Protons accelerated in the target normal sheath acceleration regime by a femtosecond laser. Phys. Rev. Accel. Beams.

[8] Hussain M., Naqvi R.A., Abbas N., Khan S.M., Nawaz S., Hussain A., Zahra N., Khalid M.W. (2020). Ultra-High-Molecular-Weight-Polyethylene (UHMWPE) as a Promising Polymer Material for Biomedical Applications: A Concise Review. Polymers.

[9] Candadai A.A., Weibel A.J., Marconnet A.M. (2020). Thermal Conductivity of Ultrahigh Molecular Weight Polyethylene: From Fibers to Fabrics. ACS Appl. Polym. Mater..

[10] Cutroneo M., Hnatowicz V., Mackova A., Malinsky P., Miksova R., Ceccio G., Maly J., Smejkal J., Štofik M., Havranek V. (2022). Ion Lithography of Single Ions Irradiation for Spatially Regular Arrays of Pores in Membranes of Polyethylene Terephthalate. Nanomaterials.

[11] Valenza A., Visco A.M., Torrisi L., Campo N. (2004). Characterization of ultra-high-molecular weight polyethylene (UHMWPE) modified by ion Implantation. Polym. J..

[12] Vancha A.R., Govindaraju S., Parsa K.V., Jasti M., González-García M., Ballestero R.P. (2004). Use of polyethyleneimine polymer in cell culture as attachment factor and lipofection enhancer. BMC Biotechnol..

[13] Dangsheng X. (2005). Friction and wear properties of UHMWPE composites reinforced with carbon fiber. Mater. Lett..

[14] Senna C.M., Santos D., Geraldi T.S. (2018). Coatings for saltwater Pipelines. IRJAES.

[15] Hermann K.P., Geworski L., Muth M., Harder D. (1985). Polyethylene-based water-equivalent phantom material for x-ray dosimetry at tube voltages from 10 to 100 kV. Phys. Med. Biol..

[16] Torrisi L. (2014). Ion Acceleration and D-D Nuclear Fusion in Laser-Generated Plasma from Advanced Deuterated Polyethylene. Molecules.

[17] Visco A.M., Brancato V., Torrisi L., Cutroneo M. (2014). Employment of Carbon Nano Materials for the Welding of Polyethylene Joints with a Nd:YAG Laser. Int. J. Polym. Anal..

[18] Chmutin I., Novokshonova L., Brevnov P., Yukhayeva G., Ryvkina N. (2017). Electrical properties of UHMWPE/graphite nanoplates composites obtained by in-situ polymerization method. Polyolefins J..

[19] Zhang Z., Zhao Y., Li H., Percec S., Yin J., Ren F. (2019). Nanoparticle-Infused UHMWPE Layer as Multifunctional Coating for High-Performance PPTA Single Fibers. Sci. Rep..

[20] Xie K., Wang W., Li Y., Xu M., Han Z., Zhang Y., Gao W. (2022). Study on structure-performance relationship of RGO enhanced polypropylene composites with improved atomic oxygen resistance. Compos. Part B Eng..

[21] Gao W., Ma Y., Zhang Y., Chen Q., Chen H., Zhu B., Jia J., Huang A., Xie K., Bai Y. (2018). Architecture & functionalization evolution of RGO affect physicomechanical properties of polyolefin/RGO composites. Compos. Part A Appl. Sci. Manuf..

[22] Fung M., Bowsher J.G., Van Citters D.W. (2018). Variation of mechanical properties and oxidation with radiation dose and source in highly crosslinked remelted UHMWPE. J. Mech. Behav. Biomed. Mater..

[23] Visco A.M., Brancato V., Cutroneo M., Torrisi L. (2014). Nd:Yag laser irradiation of single lap joints made by polyethylene and polyethylene doped by carbon nanomaterials. J. Phys. Conf. Ser..

[24] Visco A.M., Torrisi L., Campo N., Picciotto A. (2010). Comparison of Surface modifications induced by ion implantation in UHMWPE. Int. J. Polym. Anal..

[25] Lorusso A., Nassisi V., Velardi L., Torrisi L., Margarone D., Mezzasalma A. (2008). Characteristic modification of UHMWPE by laser-assisted ion implantation. Rad. Eff. Def. Solids.

[26] Torrisi L., Auditore L., Barnà R.C., De Pasquale D., Emanuele U., Loria D., Trifirò A., Trimarchi M., Campo N., Visco A. (2007). Measurements of gas desorption from polyethylene-UHMWPE irradiated by 5 MeV Electrons. Rad. Eff. Def. Solids.

[27] Visco A.M., Brancato V., Campo N., Torrisi L., Caridi F., Cutroneo M. (2013). Modification in polyethylene-iron oxide joints induced by laser irradiation. Appl. Surf. Sci..

[28] Alnefaie K.A. (2014). Strength and modulus of carbon nanotubes under a tensile load. J. Mech. Behav. Biomed. Mater..

[29] Samad M.A., Sinha S.K. (2011). Mechanical, thermal and tribological characterization of a UHMWPE film reinforced with carbon nanotubes coated on steel. Tribol. Int..

[30] Galtieri G., Visco A., Nocita D., Torrisi L., Ceccio G., Scolaro C. (2016). Polyethylene laser welding based on optical absorption variations. J. Instrum..

[31] Al-Saleh M.H., Jawad S.A., El Ghanem H.M. (2014). Electrical and dielectric behaviors of dry-mixed CNT/UHMWPE Nanocomposites. High Perform. Polym..

[32] Visco A.M., Torrisi L., Scolaro C. (2016). Effect of the filler amount on the optical absorption properties and the surface features of polymeric joints based on biomedical UHMWPE welded by a Nd:YAG laser. J. Thermoplast. Compos. Mater..

[33] Zabolotnov A.S., Brevnov P.N., Akul’shin V.V., Novokshonova L.A., Doronin F.A., Evdokimov A.G., Nazarov V.G. (2018). The Wear Resistance of Composite Materials Based on Ultra-High-Molecular-Weight Polyethylene with Fillers of Various Types. Polym. Sci. Ser. D.

[34] Drakopoulos S.X., Psarras G.C., Ronca S. (2021). Oriented ultra-high molecular weight polyethylene/gold nanocomposites: Electrical conductivity and chain entanglement dynamics. Express Polym. Lett..

[35] Scolaro C., Visco A., Torrisi L., Pedullà E. (2018). White-white polymer joints made with different laser absorbing nano fillers: Physical-mechanical features. AIP Conf. Proc..

[36] Kang X., Yao C., Qiao L., Ge G., Feng P. (2017). Processing and Mechanical Properties of Ultra-high Molecular Weight Polyethylene Reinforced by Silver Nanoparticles. Polym. Polym. Compos..

[37] Kumar V., Alam M.N., Park S.S. (2022). Soft Composites Filled with Iron Oxide and Graphite Nanoplatelets under Static and Cyclic Strain for Different Industrial Applications. Polymers.

[38] Merck Carbon Nanotube, Multi-Walled. https://www.sigmaaldrich.com/IT/it/product/aldrich/698849.

[39] Merck Fe2O3 Nanoparticles. https://www.sigmaaldrich.com/IT/it/search/fe2o3-nanoparticles?focus=products&page=1&perpage=30&sort=relevance&term=fe2o3%20nanoparticles&type=product_name.

[40] Sigle W. (2005). Analytical transmission electron microscopy. Annu. Rev. Mater. Res..

[41] De Geyter N., Morent R., Leys C. (2008). Surface characterization of plasma-modified polyethylene by contact angle experiments and ATR-FTIR spectroscopy. Surf. Interface Anal..

[42] Ozkahraman B., Tamahkar Irmak E. (2007). Carbon nanotube based polyvinylalcohol-polyvinylpyrolidone nanocomposite hydrogels for controlled drug delivery applications. Anadolu Univ. J. Sci. Technol. A-Appl. Sci. Eng..

[43] Sbai K., Rahmani A., Chadli H., Bantignies J.L., Hermet P., Sauvajol J.L. (2006). Infrared Spectroscopy of Single-Walled Carbon Nanotubes. J. Phys. Chem. B.

[44] Branca C., Frusteri F., Magazu` V., Mangione A. (2004). Characterization of Carbon Nanotubes by TEM and Infrared Spectroscopy. J. Phys. Chem. B.

[45] Farahmandjou M., Soflaee F. (2015). Synthesis and Characterization of α-Fe_2_O_3_ Nanoparticles by Simple Co-Precipitation Method. Phys. Chem. Res..

[46] Visco A., Scolaro C., Terracciano T., Montanini R., Quattrocchi A., Torrisi L., Restuccia N. (2018). Static and dynamic characterization of biomedical polyethylene laser welding using biocompatible nanoparticles. EPJ Web Conf..

[47] Mott N.F., Davis E.A. (1971). Electronic Processes in Non-Crystalline Materials.

[48] Abdul-Kader A.M. (2013). The optical band gap and surface free energy of polyethylene modified by electron beam Irradiations. J. Nucl. Mater..

[49] Sabet M., Soleimani H. (2014). Mechanical and electrical properties of low density polyethylene filled with carbon nanotubes. IOP Conf. Ser. Mater. Sci. Eng..

[50] Mizuno S., Yao H. (2021). On the electronic transitions of α-Fe_2_O_3_ hematite nanoparticles with different size and morphology: Analysis by simultaneous deconvolution of UV–vis absorption and MCD spectra. J. Magn. Magn. Mater..

[51] Vakhshouri A.R., Azizov A., Aliyeva R., Bagirova S. (2012). Synthesis, Structure, and Thermo-Physical Properties of Fe_2_O_3_.Al_2_O_3_ and Polyethylene Nanocomposites. J. Appl. Polym. Sci..

[52] Oleiwi J.K., Anaee R.A., Radhi S.H. (2018). Roughness, wear and thermal analysis of UHMWPE nanocomposites as acetabular cup in hip joint replacement. Int. J. Mech. Prod. Eng. Res. Dev. (IJMPERD).

